# Effectiveness of Extracorporeal Shock Wave Therapy in Treatment of Spasticity of Different Aetiologies: A Systematic Review and Meta-Analysis

**DOI:** 10.3390/jcm13051323

**Published:** 2024-02-26

**Authors:** Iris Otero-Luis, Iván Cavero-Redondo, Celia Álvarez-Bueno, Arturo Martinez-Rodrigo, Carlos Pascual-Morena, Nerea Moreno-Herráiz, Alicia Saz-Lara

**Affiliations:** 1 Health and Social Research Center, Universidad de Castilla-La Mancha, 16071 Cuenca, Spain; iris.otero@ulcm.es (I.O.-L.); celia.alvarezbueno@uclm.es (C.Á.-B.); carlos.pascual@uclm.es (C.P.-M.); nerea.moreno@uclm.es (N.M.-H.); alicia.delsaz@uclm.es (A.S.-L.); 2 Facultad de Ciencias de la Salud, Universidad Autonoma de Chile, Talca 3460000, Chile; 3 Universidad Politécnica y Artística del Paraguay, Asunción 2024, Paraguay; 4 Departamento de Sistemas Informáticos (DSI), Facultad de Comunicación de Cuenca, University of Castilla-La Mancha, 16071 Cuenca, Spain; arturo.martinez@uclm.es

**Keywords:** extracorporeal shock wave therapy, spasticity, children with cerebral palsy, adults with chronic stroke, upper limbs, lower limbs

## Abstract

**Background**: Spasticity is a motor disorder characterised by exaggerated movements of the tendons and accompanied by hyperreflexia and hypertonia. Extracorporeal shock wave therapy (ESWT) is used as a treatment for spasticity, although more evidence is needed on the effectiveness of this therapy in the treatment of spasticity. Therefore, the aim of this study was to assess the effectiveness ESWT in the treatment of upper and lower limbs spasticity in both children and adults through different aetiologies. **Methods**: A systematic search was performed in different databases from inception to December 2023. Random-effects meta-analysis was used to estimate the efficacy of ESWT on spasticity using the Modified Ashworth Scale. **Results**: Sixteen studies were included in the systematic review and meta-analysis. The effect of ESWT on spasticity measured with the Modified Ashworth Scale shows a significant decrease in spasticity in the upper limbs and in the lower limbs in adults with chronic stroke and in children with cerebral palsy, is more effective immediately after application, and maintains its effect up to 12 weeks post treatment. **Conclusions**: These findings are important for clinical practice since they show evidence that ESWT is effective in reducing spasticity in both children and adults.

## 1. Introduction

Spasticity is defined as a motor disorder characterised by a velocity-dependent increase in the tonic muscle stretch reflex (myotatic), resulting in exaggerated tendon movements and accompanied by hyperreflexia and hypertonia [1]. This is due to neuronal hyperexcitability, which is a sign of upper motor neuron syndrome [2]. Individuals with spasticity may present with symptoms such as multijoint spasms, simultaneous contraction of agonist and antagonist muscles, and dystonia or abnormal postures [1]. Spasticity can be due to different aetiologies, affecting 20–40% of stroke patients [2], more than 80% of cerebral palsy (CP) patients [3], 13–20% of cranioencephalic trauma patients, 60–78% of spinal cord trauma patients and 60–90% of multiple sclerosis patients [3]. Currently, there are no objective tools available to measure the degree of spasticity [4], so scales are used to assess this disorder, such as the Ashworth scale, the Tardieu scale, the Penn scale, or pendulum test, among others [4]. In particular, the Modified Ashworth Scale (MAS) evaluates the resistance offered by a muscle during passive movement, ranging from zero (normal muscle tone) to four (stiff rigid in flexion or extension) [5].

There are many options for treating spasticity, with treatment being individualised and adapted to the characteristics of the patient. Therapeutic options include pharmacological and nonpharmacological treatments [1]. Pharmacological treatment may be used to control spasticity in combination with nonpharmacological treatment, which focuses on achieving a greater degree of functionality by acting on soft tissues and joints to prevent stiffness, contractures, deformity, and pain [1]. Nonpharmacological techniques include physiotherapy, assessment of orthoses and assistive devices, application of shock waves, or radiofrequency [6]. In recent years, extracorporeal shock wave therapy (ESWT) has been used, which can be used alone or in combination with other treatments, such as botulinum toxin [3]. ESWT is a promising intervention for spasticity, as it disrupts the link between actin and myosin, thereby reducing connective tissue stiffness. The physical effects of ESWT include cavitation and microtrauma, as well as potential neurological impacts that alter neurotransmitter expression [7]. In addition, ESWT influences vascularisation, inflammatory processes and connective tissue remodelling [8]. The integration of clinical evidence supports the efficacy of ESWT in the treatment of spasticity, underscoring the need for further research to refine treatment protocols and optimise the therapeutic potential of ESWT for people suffering from spasticity [9]. This therapeutic approach, classified as focused or radial shockwave, represents a nuanced strategy to modulate spasticity through targeted mechanical interventions [10]. ESWT is defined as a sequence of single sonic pulses, characterised by a high pressure peak (greater than 100 MPa), a rapid pressure increase (<10 ns), and a short duration (10 ms), transmitted by a suitable generator to a specific area of the body [5].

There are several previous systematic reviews and meta-analyses examining the treatment of spasticity with extracorporeal shock waves. Many of these studies focus on the efficacy of this therapy in post-stroke patients [5,11,12,13,14]. These authors agree that ESWT effectively improves spasticity [5,11,12,13,14] in the short and long term [5,12,13], being a non-invasive therapy [14]. Improvements are observed in passive movement [5], range of motion [12,13,14], motor function [3,11,12] and functional independence [4]. Some studies report pain relief [3,13,14]. Cavanas-Valdés R et al. argue that to ensure efficacy, the area to be treated should be identified by ultrasound or radiography to obtain a more favourable therapeutic effect and to avoid damaging the surrounding tissue [12]. Other studies focus on this therapy applied to people with cerebral palsy [15,16]. These authors argue that this therapy is safe, non-invasive, and effective and improves spasticity and motor function [15], reducing the MAS scale and increasing the quality of life of children with CP [16]. Our study aims to assess the duration of the effect of ESWT once the treatment is completed.

Although there is evidence of the efficacy of ESWT in the treatment of spasticity, it is necessary to assess whether this therapy is effective in different aetiologies, both in children and adults. Therefore, the objectives of this systematic review and meta-analysis were (i) to estimate the efficacy of ESWT in the treatment of upper limb (UL) and lower limb (LL) spasticity, (ii) to estimate the efficacy of ESWT in the treatment of spasticity in both children with CP and adults with chronic stroke, and (iii) to estimate the efficacy of ESWT in the treatment of spasticity for postintervention time periods.

## 2. Materials and Methods

This systematic review and meta-analysis was conducted according to the Preferred Reporting Items for Systematic Reviews and Meta-Analyses (PRISMA) guidelines [17] and the recommendations of the Cochrane Handbook [18]. This study was registered in PROSPERO (identification number: CRD42023436889).

### 2.1. Search Strategy

For this systematic review and meta-analysis, we searched the PubMed, Scopus, Web of Science, and Cochrane Library databases from inception to December 2023. The search terms included adults, children, extracorporeal shock wave therapy, spasticity, cerebral palsy, spastic paraplegia, chronic stroke, randomised controlled trial, and RCT, combined with Boolean operators (AND, OR) according to the PICO (population, intervention, comparator, outcome) strategy, to identify primary studies evaluating the effect of ESWT in the treatment of spasticity (Supplementary Table S1).

In addition, previous systematic reviews and meta-analyses and reference lists of retrieved articles were examined to identify any additional relevant studies.

### 2.2. Inclusion and Exclusion Criteria

Inclusion criteria were as follows: (i) population: subjects (both children [<18 years] and adults [>18 years]) with spasticity of different aetiologies; (ii) intervention: ESWT (radial and focused); (iii) comparator: control group (CG); (iv) outcome: spasticity assessed with the MAS scale; and (v) study design: randomised controlled trials (RCTs) and crossover clinical trials (CCTs). The following were excluded: (i) articles written in languages other than English or Spanish; (ii) review articles, editorials or case reports; and (iii) studies combining the treatment under investigation with other treatments.

### 2.3. Data Extraction

An ad hoc table was created with the following data extracted from the included studies: (1) reference (first author and year of publication), (2) country in which the study data were collected, (3) study design (RCTs and CCTs), (4) population characteristics (sample size, mean age, and type of population), (5) intervention characteristics (frequency, energy, and impact of ESWT), and (6) outcome variable (measurement method and mean value of reduction of the MAS scale).

### 2.4. Quality Assessment

The quality of the included trials was assessed using the Cochrane Collaboration’s tool for assessing risk of bias (RoB2) [19]. This tool assesses the risk of bias based on five domains: randomisation process, deviations from interventions, missing outcome data, outcome measurement, and selection of the reported outcome. Overall bias was rated as “low risk of bias” when all domains were classified as “low risk”, “some concerns” when there was at least one domain classified as “some concern”, and “high risk of bias” when there was at least one domain classified as “high risk” or several domains classified as “some concerns”.

Two researchers (I.O.-L. and A.S.-L.) independently performed study selection, data extraction, and quality assessment of the included RCTs. Disagreements were resolved by consensus or by a third reviewer (I.C.-R.).

### 2.5. Statistical Analysis and Data Synthesis

The DerSimonian and Laird random-effects method [20] was used to calculate pooled estimates of mean differences (MDs) and their respective 95% confidence intervals (95% CIs) to assess the effect of ESWT on spasticity according to upper limb (UL) or lower limb (LL). In addition, a meta-analysis was performed in the intervention group (IG) for the time periods (i.e., immediately post intervention, one week postintervention, 2–4 weeks postintervention, 5–12 weeks postintervention, and more than 12 weeks postintervention) to assess the duration of efficacy of ESWT on spasticity. Meta-analysis required at least five studies for reach exposure group [21]. Heterogeneity was assessed using the *I*^2^ statistic [22], which ranges from 0% to 100%. Depending on the *I*^2^ values, heterogeneity was considered not important (0% to 30%), moderate (30 to 60%), substantial (60 to 75%), or considerable (75 to 100%). Corresponding *p* values were also considered. The *p* value of heterogeneity was also considered and was considered statistically significant if *p* < 0.05.

A sensitivity analysis (systematic reanalysis by eliminating studies one at a time) was performed to assess the robustness of the summary estimates. In addition, a subgroup analysis was performed according to population type (children with CP and adults with chronic stroke). Meta-regression models were applied for mean age, percentage of females, treatment duration, frequency, energy, and impact of the intervention on UL and LL to assess whether the effect of ESWT on spasticity could be modified. Finally, publication bias was assessed using Egger’s asymmetry test [23]. A level of <0.1 was used to determine whether publication bias might be present.

All statistical analyses were performed using STATA SE software, version 15 (StataCorp, College Station, TX, USA).

## 3. Results

### 3.1. Baseline Characteristics

**Figure 1:**
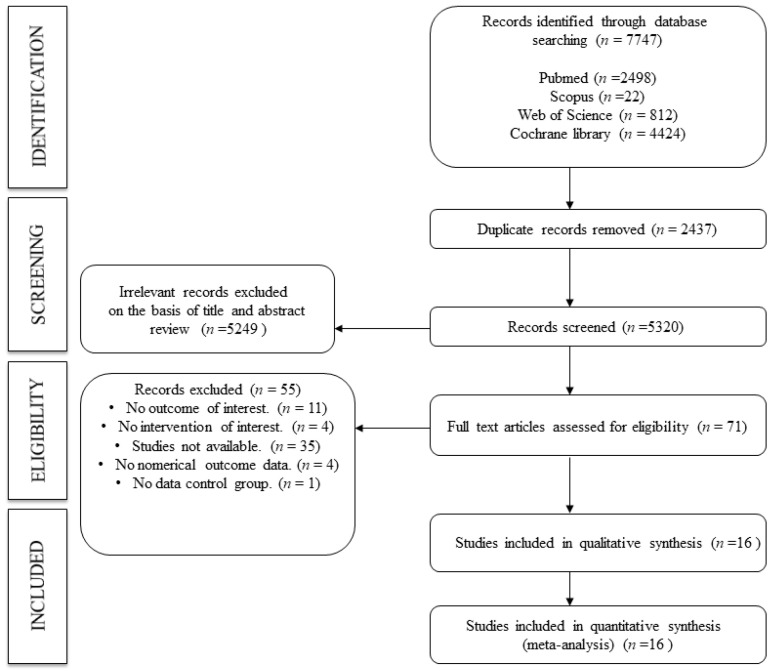
Flowchart: search strategy.

The search strategy recovered a total of 7747 studies from four databases (PubMed, *n* = 2498; Scopus, *n* = 22; Web of Science, *n* = 812; and Cochrane library, *n* = 4424), of which 71 were assessed in full text. 55 studies were excluded for the following reasons: no outcome of interest (*n* = 11), no intervention of interest (*n* = 4), studies not available (*n* = 35), no numerical outcome data (*n* = 4), and no data control group (*n* = 1). Finally, we included 16 studies in both the systematic review and meta-analysis. (Figure 1).

The studies included in this systematic review were published between 2005 and 2022. All included studies were RCTs [24,25,26,27,28,29,30,31,32,33,34,35,36,37], except for two, which were CCTs [37,38]. Study participants (704) were both children and adults (aged between 26.9 ± 13.1 months and 66.9 ± 4.9 years) with spasticity caused by different aetiologies, such as CP [23,24,28,31,39], chronic stroke [26,27,29,30,32,33,34,35,36,38], or multiple sclerosis [37]. The length of the studies ranged from one to sixteen weeks. The minimum frequency was 4 Hz, the maximum was 10 Hz, the minimum energy was 0.03 mJ/mm^2^, the maximum was 0.84 mJ/mm^2^, the minimum impact was 800 times per muscle, and the maximum was 4000. All included studies assessed spasticity using the MAS scale, from which the mean reduction score was extracted. The characteristics of the included studies are shown in Table 1.

### 3.2. Quality Assessment

The methodological quality of the studies was assessed using the Cochrane Collaboration’s tool for assessing the risk of bias of RCTs (ROB2) [19]. Of the included studies, 66.7% of the studies had a “low risk of bias”, and 33.3% of the studies had “some concerns” (Supplementary Figures S1 and S2).

### 3.3. Effect of Extracorporeal Shock Wave Therapy (ESTW) on Spasticity

**Figure 2:**
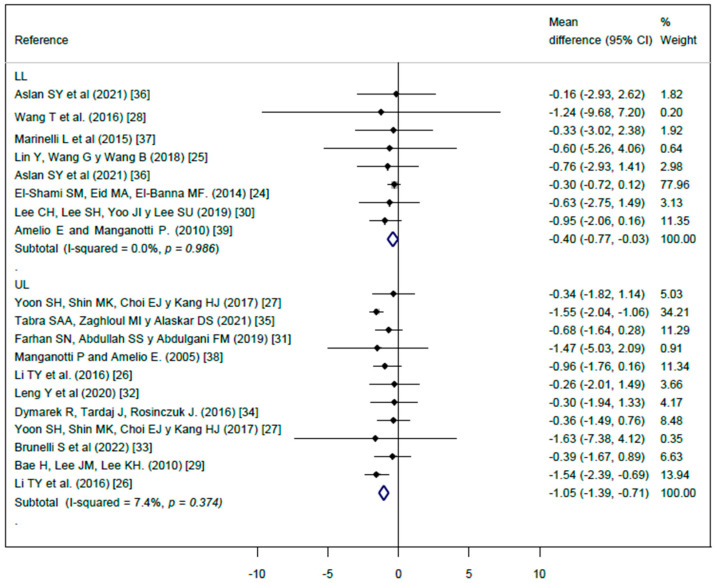
Forest plot for the effect of extracorporeal shock wave therapy (ESWT) on upper limb (UL) spasticity and lower limb (LL) spasticity [24,25,26,27,28,29,30,31,32,33,34,35,36,37,38,39].

The effect of ESWT on spasticity measured by the MAS scale showed a significant decrease in spasticity in ULs (MD: −1.05; 95% CI: −1.39, −0.71), with nonsignificant heterogeneity (*I*^2^ = 7.7%), and in LLs (MD: −0.40; 95% CI: −0.77, −0.03), with nonsignificant heterogeneity (*I*^2^ = 0.0%) (Figure 2).

### 3.4. Effect of Extracoporeal Shock Wave Therapy (ESTW) on Spasticity for Time Periods after Intervention

**Figure 3:**
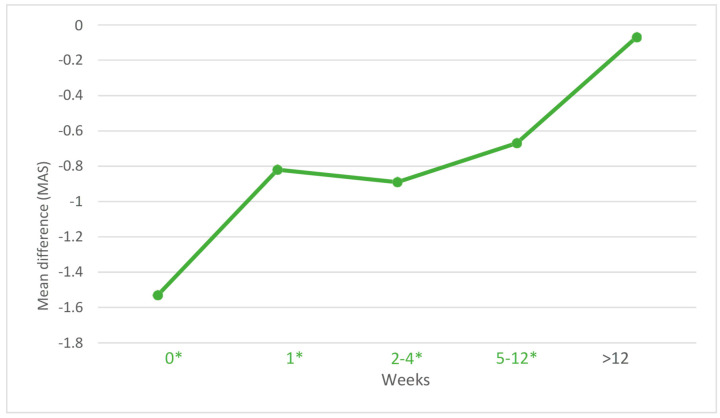
Effect of extracorporeal shock wave therapy (ESWT) on spasticity according to the duration of the effect of the intervention. * *p* < 0.05.

The effect of ESWT on spasticity showed a significant decrease in the MAS score immediately after the intervention (MD: −1.35; 95% CI: −1.89, −0.81), with considerable heterogeneity (*I*^2^ = 85.8%), at one week postintervention (MD: −0.79; 95% CI: −1.16, −0.42), with substantial heterogeneity (*I*^2^ = 76.6%), between 2 and 4 weeks postintervention (MD: −0.92; 95% CI: −1.25, −0.60), with substantial heterogeneity (*I*^2^ = 74.8%), and between 5 and 12 weeks postintervention (MD: −0.75; 95% CI: −1.22, −0.27), with substantial heterogeneity (*I*^2^ = 86.4%). After 12 weeks postintervention, there were no significant results for the efficacy of ESWT in the treatment of spasticity (MD: −0.47; 95% CI: −1.30, 0.35) (Figure 3).

### 3.5. Sensitivity Analysis

The pooled MD estimate for the effect of ESWT on spasticity was not significantly changed (in magnitude or direction) when analyses of individual study data for ULs and LLs were removed one at a time.

### 3.6. Subgroup Analysis and Meta-Regression Models

The subgroup analysis according to population type (children [<18 years] with CP [242 subjects] and adults [>18 years] with chronic stroke [462 subjects]) showed a significant decrease in spasticity on the MAS scale in adults with chronic stroke (MD: −1.11; 95% CI: −1.42, −0. 80), with nonsignificant heterogeneity (*I*^2^ = 0.0%, *p* = 0.544), and in children with CP (MD: −0.43; 95% CI: −0.79, −0.06), with nonsignificant heterogeneity (*I*^2^ = 0.0%, *p* = 0.690) (Supplementary Table S2).

Meta-regression models showed that in ULs, both treatment duration (*p* = 0.038) and energy (*p* = 0.026) could modify the effect of ESWT on spasticity. Specifically, as duration and energy of treatment increase, we observed a decrease in the MAS scores, indicating an improvement in spasticity. These variables have been identified as confounders, contributing to the heterogeneity of the study (Supplementary Table S3).

### 3.7. Publication Bias

Finally, evidence of publication bias was observed in the UL by Egger’s test for ESWT on spasticity (*p* = 0.062) (Supplementary Figure S3). However, no publication bias was observed in the LL (*p* = 0.237) (Supplementary Figure S4).

## 4. Discussion

This systematic review and meta-analysis shows evidence of the effectiveness of ESWT in reducing spasticity in ULs, being effective up to 12 weeks postintervention. In addition, our results show that this therapy is more effective in post-stroke adults than in children with CP, presenting a significant decrease in the MAS scale in both subgroups. On the other hand, the duration of treatment and energy have a direct relationship with the reduction of the MAS scale in ULs, i.e., the shorter the duration of treatment and the lower the energy, the greater the reduction in the MAS scale, meaning a greater effect of the therapy.

According to a previous systematic review, ESWT is considered a good treatment for spasticity, improving motor function and impairment, thus reducing pain and improving functional independence, even with a single session [3]. Another previous systematic review and meta-analysis indicates that this therapy reduces the MAS scale, improving patients’ quality of life, while being minimally invasive [17]. Our findings support this previous evidence on the effectiveness of ESWT in spasticity, i.e., being effective in ULs, in post-stroke patients, and in children with CP and showing a greater effect immediately after therapy, which is maintained up to 12 weeks post treatment.

ESWT is a sequence of single sonic pulses characterized by a high peak pressure (>100 MPa), a rapid pressure rise (less than 10 ns), and a short duration (10 ms). These waves are transmitted through a suitable generator and applied to a specific area of the body. The energy used in this therapy ranges between 0.003 and 0.89 mJ/mm^2^ [5]. According to previous evidence, this therapy is applied to patients with post-stroke spasticity [26,27,29,30,32,33,34,35,36,38] and to patients with CP [23,24,28,31,39], regardless of whether they are children or adults; our results show that the therapy is effective in both groups, while being more effective in post-stroke adults.

According to the studies reviewed, ESWT is a non-invasive [38,39], safe [28,34,36,38,39], painless, and uncomplicated treatment [39]. In addition, it reduces muscle flexor tone, thus improving spasticity [26,32,38,39]. One RCT reports that this therapy is effective in treating post-stroke spasticity regardless of the site of application (muscle belly or myotendinous junction) [27]. However, another RCT reports that the treatment effect is greater at the myotendinous junction, indicating that age, initial severity of spasticity, and disease duration are not significantly related to the effectiveness of the therapy with respect to spasticity [29]. According to another study, repeated sessions of this therapy produce a more lasting and noticeable effect and improve functional motor function [26]. Another trial reports that this therapy seems to prevent the progression of spasticity to higher degrees and reduces the use of oral antispasmodics [33]. Despite the variation in populations studied, therapy application locations, and effects observed, there is evidence supporting the effectiveness of ESWT in the treatment of spasticity.

There are some limitations to be taken into account in this study. First, the results should be interpreted with caution since there is heterogeneity due to differences between populations (age or aetiology); however, subgroup analyses and meta-regressions were performed to mitigate heterogeneity. Secondly, the sample sizes are small because the prevalence of diseases with spasticity is low; therefore, it is difficult to find subjects for ESWT. Thirdly, this therapy depends on frequency (Hz), energy (mJ/mm^2^), and impact (number of shots per muscle), so there are differences at the study and patient levels in the included studies. Fourth, only the MAS scale was included to assess spasticity [40] and is administered by a professional who conducts a direct physical assessment of the patient, and there may be evaluator and patient bias. Fifth, due to the lack of studies on certain aetiologies, such as multiple sclerosis, a subgroup analysis could not be performed to evaluate the effect of the intervention in this type of population. Therefore, RCTs of high methodological quality and with large sample sizes in different types of populations (children and adults) and according to aetiology are needed to elucidate the effect of ESWT in the treatment of spasticity.

## 5. Conclusions

In summary, this systematic review and meta-analysis shows the effectiveness of extracorporeal shock wave therapy in reducing spasticity in ULs, post-stroke adults, and children with CP. This therapy is most effective immediately after application, maintaining effectiveness up to 12 weeks post treatment. These findings are important for clinical practice since they show evidence that ESWT is effective for the treatment of spasticity, which is beneficial and safe, since it is not painful or invasive, does not present complications, and allows a reduction spasticity, thus increasing the patient’s quality of life. Even so, more RCTs with a larger sample size are needed to demonstrate the efficacy of ESWT and to be able to generalize its use in spasticity in daily clinical practice.

## Figures and Tables

**Table 1:** Characteristics of the included studies.

Reference	Country	Study Design	Population Characteristics				Intervention Characteristics					Outcome Variable: Spasticity	
			Sample Size (*n* Women)	Age (Years)	Population Type	Baseline MAS	Area of Application	Measurement Weeks	Frecuency (Hz)	Energy (mJ/mm^2^)	Impact (Shots per Muscle)	Measurement Method	MAS Scale Reduction (MD ± SD)
Manganotti, P. and Amelio, E. (2005) [38]	Italy	CCT	20 (9)	38–76	Chronic stroke	EG: 3.28 ± 2.04 CG: 3.28 ± 2.04	Wrist flexor Finguer flexor	Inmediatly after therapy, weeks 1, 4, and 12	NR	0.030	800–1500	MAS	EG: −1.59 ± 7.397 CG: −0.72 ± 2.661
Amelio, E. and Manganotti, P. (2010) [39]	Italy	CCT	12 (6)	8 ± 2.31	CP	EG: 3.3 ± 0.49 CG: 3.3 ± 0.49	Plantar flexors	Inmediatly after therapy, weeks 1, 4, and 12	NR	0.030	1500	MAS	EG: −1.15 ± 1.750 CG: −0.2 ± 0.620
Bae, H., Lee, J.M., Lee, K.H. (2010) [29]	Republic of Korea	RCT	32 (12)	EG: 56.7 ± 12.4 CG: 53.4 ± 16.8	Chronic stroke	EG: 2.9 ± 0.3 CG: 2.6 ± 0.5	Elbow flexors	Inmediatly after therapy, weeks 1 and 4	4	0.12	1200	MAS	EG: −0.42 ± 1.713 CG: −0.03 ± 1.224
El-Shami, S.M., Eid, M.A., El-Banna, M.F. (2014) [24]	Egypt	RCT	30 (12)	EG: 6.93 ± 0.8 CG: 6.8 ± 0.7	CP	EG: 2.34 ± 0.48 CG: 2.27 ± 0.92	Ankle plantar flexor	Week 12	5	0.030	1500	MAS	EG: −0.71 ± 0.531 CG: −0.41 ± 0.600
Marinelli, L. et al. (2016) [37]	Italy	RCT	68 (38)	EG: 51.74 ± 11.29 CG: 51 ± 13.17	Multiple sclerosis	EG: 2.68 ± 0.77 CG: 2.56 ± 0.92	Ankle extensor muscles and Achiles tendon	Inmediatly after therapy, weeks 1 and 4	4	NR	2000	MAS	EG: −0.44 ± 5.147 CG: −0.11 ± 6.024
Dymarek, R., Tardaj, J., Rosinczuk, J. (2016) [34]	Poland	RCT	60 (26)	EG: 61.46 ± 12.74 CG: 60.87 ± 9.51	Chronic stroke	EG: 1.66 ± 1.65 CG: 1.68 ± 1.93	Elbow joint Finger joints Radio-carpal joints	Inmediatly after therapy	5	0.030	1500	MAS	EG: −0.317 ± 2.321 CG: −0.015 ± 2.768
Li, T.Y. et al. (2016) [26]	China	RCT	60 (19)	EG1: 55.35 ± 3.05 EG2: 56.80 ± 3.00 CG: 55.95 ± 2.64	Chronic stroke	EG1: 3.07 ± 0.89 EG2: 3.10 ± 0.68 CG: 2.65 ± 0.03	Wrist Hand	Inmediatly after therapy, weeks 1, 4, 8, 12, and 16	5	NR	1500–4000	MAS	EG1: −1.51 ± 1.825 EG2: −0.93 ± 1.688 CG: 0.03 ± 0.491
Wang, T. et al. (2016) [28]	China	RCT	86 (22)	EG: 26.9 ± 13.1 month CG: 27.0 ± 14.2 month	CP	EG: 2.09 ± 2.99 CG: 2.1 ± 2.99	Plantar flexor muscles	Weeks 4 and 12	8	0.03	1500	MAS	EG: −1.35 ± 18.415 CG: −0.11 ± 15.686
Yoon, S.H., Shin, M.K., Choi, E.J. and Kang, H.J. (2017) [27]	Republic of Korea	RCT	124 (5)	EG1: 58.7 ± 15.7 EG2: 63.1 ± 11.8 CG: 63.4 ± 13.8	Chronic stroke	EG1: 2.84 ± 3.59 EG2: 2.86 ± 2.43 CG: 2.52 ± 3.13	Elbow flexor	Week 1	5	0.068–0.093	1500	MAS	EG1: −0.34 ± 4.896 EG2: −0.36 ± 3.688 CG: 0 ± 0.667
Lin, Y., Wang, G. and Wang, B. (2018) [25]	China	RCT	82	EG: 7.5 ± 1.3 CG: 7.9 ± 1.7	CP	EG: 4.50 ± 2.45 CG: 4.70 ± 2.45	Triceps and hamstring muscles	Weeks 2 and 4	10	NR	2000	MAS	EG: −2.050 ± 9.324 CG: −1.45 ± 11.853
Farhan, S.N., Abdullah, S.S. and Abdulgani, F.M. (2019) [31]	Irak	RCT	32 (15)	EG: 6.37 ± 1.44 CG: 6.68 ± 2.63	CP	NR	Wrist and elbow flexors	Week 8	10	0.03	800	MAS	EG: −1.06 ± 2.179 CG: −0.380 ± 1.631
Lee, C.H., Lee, S.H., Yoo, J.I. and Lee, S.U. (2019) [30]	Republic of Korea	RCT	18 (2)	EG: 50.89 ± 8.81 CG: 44.11 ± 4.07	Chronic stroke	EG: 2.22 ± 1.09 CG: 1.78 ± 0.67	Gastrocnemius muscle	Inmediatly after therapy, weeks 1 and 4	4	0.1	2000	MAS	EG: −0.52 ± 2.258 CG: 0.11 ± 1.967
Leng, Y. et al. (2020) [32]	China	RCT	27 (5)	EG: 51.14 ± 13.68 CG: 8.921 ± 10.08	Chronic stroke	EG: 2 ± 0.78 CG: 1.85 ± 0.80	Wrist Joint	Inmediatly after therapy and week 1	4	0.038	1500	MAS	EG: −0.96 ± 2.873 CG: −0.7 ± 1.082
Tabra, S.A.A., Zaghloul, M.I. and Alaskar, D.S. (2021) [35]	Egypt	RCT	40 (5)	EG: 55.70 ± 9.30 CG: 53.85 ± 10.20	Chronic stroke	EG: 3.17 ± 0.66 CG: 3.12 ± 0.66	Wrist and hand muscles	Weeks 2 and 12	15	0.25–0.84	2000–3000	MAS	EG: −1.55 ± 0.742 CG: 0 ± 0.787
Aslan, S.Y. et al. (2021) [36]	Turkey	RCT	49 (22)	EG1: 57.5 ± 14.3 EG2: 58.8 ± 10.8 CG: 60.6 ± 9.6	Chronic stroke	EG1: 2.5 ± 0.7 EG2: 2.2 ± 1 CG: 2.1 ± 0.9	Ankle plantar flexor	Weeks 2 and 6	10	NR	1500	MAS	EG1: −0.91 ± 2.787 EG2: −0.31 ± 4.306 CG: −0.15 ± 3.316
Brunelli, S. et al. (2022) [33]	Italy	RCT	32 (13)	EG: 54.80 ± 17.29 CG: 62.18 ± 16.17	Chronic stroke	EG: 1.09 ± 1.69 CG: 0.91 ± 1.94	Anterior area of forearm or arm or shoulder	Weeks 1 and 4	10	NR	2000	MAS	EG: −0.5 ± 6.471 CG: 1.13 ± 9.046

The results are shown as the mean ± standard deviation (SD). CCT: crossover clinical trial. CG: control group. CP: cerebral palsy. EG: experimental group. EG1: experimental group 1. EG2: experimental group 2. MAS: modified Ashworth scale. MD: mean difference. NR: not reported. RCT: randomised controlled trial.

## Data Availability

Data are contained within the article and supplementary materials.

## Supplementary Materials

The following supporting information can be downloaded at https://www.mdpi.com/article/10.3390/jcm13051323/s1; Table S1: Search strategy; Table S2. Subgroup analysis by aetiology of spasticity (children [<18 years] with CP and adults [>18 years] with chronic stroke) for the effectiveness of ESWT; Table S3: Meta-regression models according to mean age, percentage of women, treatment duration, frequency (Hz), energy (mJ/mm^2^), and impact (shots/muscle) for the effectiveness of ESWT; Figure S1: Quality assessment of the included studies using the Cochrane Collaboration’s tool for assessing risk of bias (RoB2); Figure S2: Quality assessment using the Cochrane Collaboration’s tool for assessing risk of bias (RoB2); Figure S3: Funnel plot for lower limbs; and Figure S4: Funnel plot for upper limbs. References [24,25,26,27,28,29,30,31,32,33,34,35,36,37,38] are cited in Supplementary Materials.
