# Supplementary figures and images for: Effectiveness of Extracorporeal Shock Wave Therapy in Treatment of Spasticity of Different Aetiologies: A Systematic Review and Meta-Analysis
Source: J Clin Med. 2024 Feb 26;13(5):1323. doi: 10.3390/jcm13051323 (PMC10932441; doi:10.3390/jcm13051323)

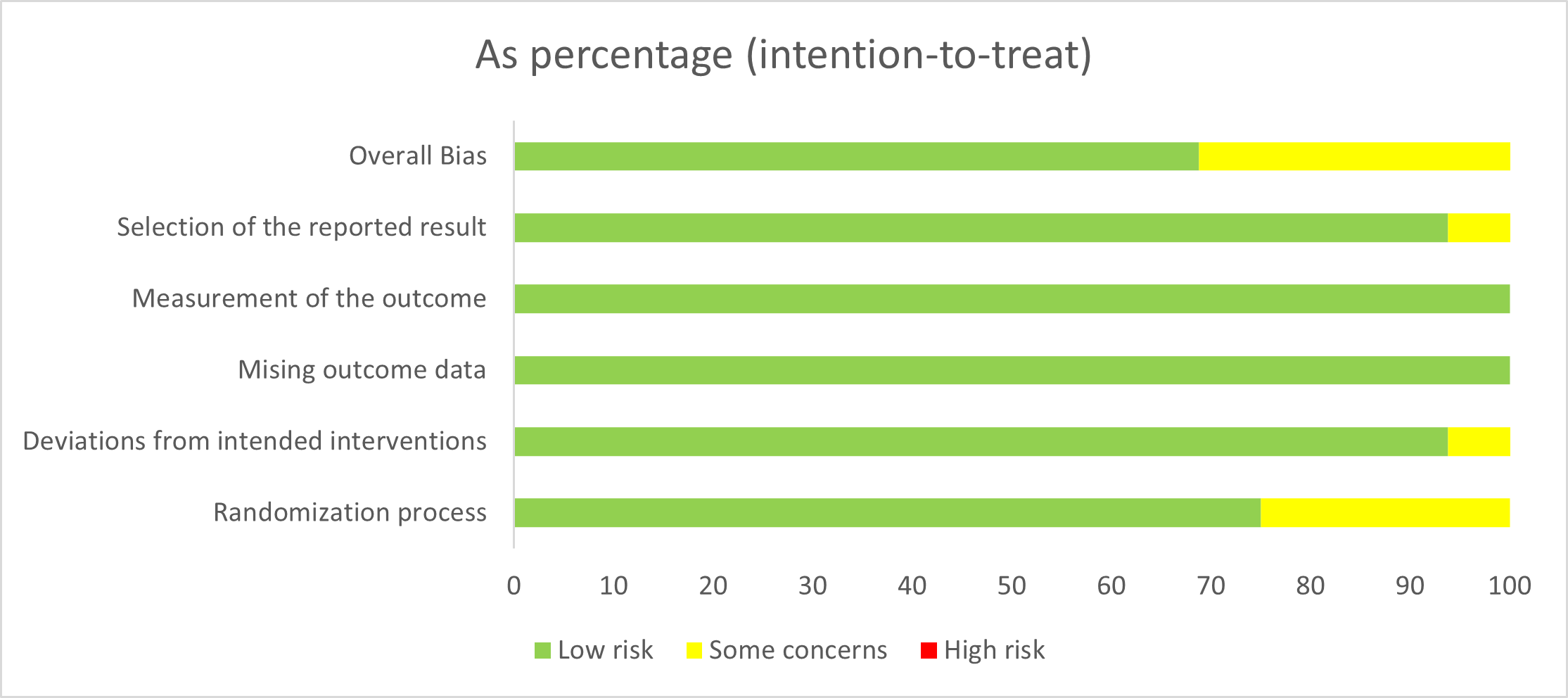

Supplement: Supplementary file 1 [file jcm-13-01323-s001.zip › Supplementary Figure S2.tif]

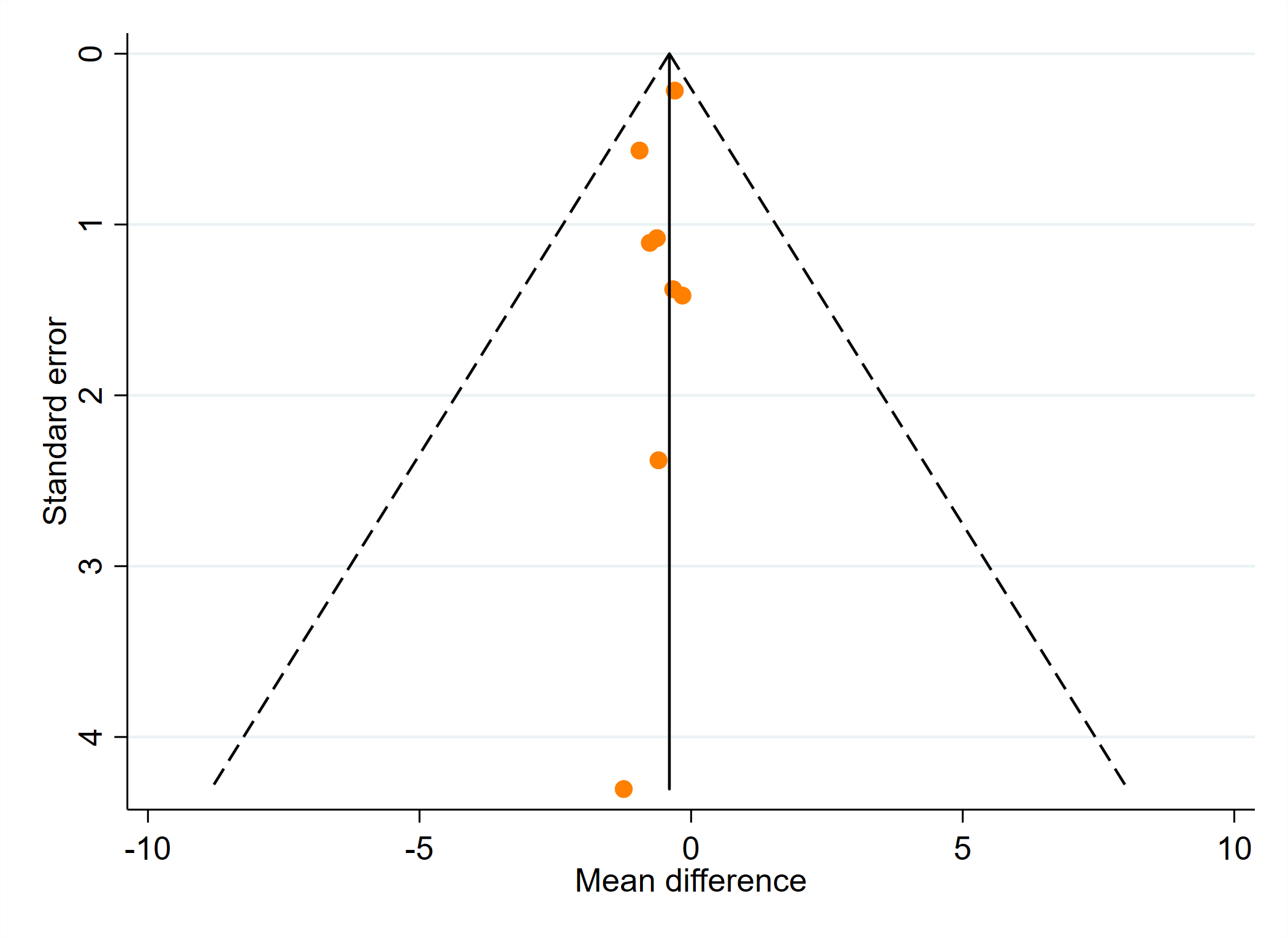

Supplement: Supplementary file 1 [file jcm-13-01323-s001.zip › Supplementary Figure S3.tif]

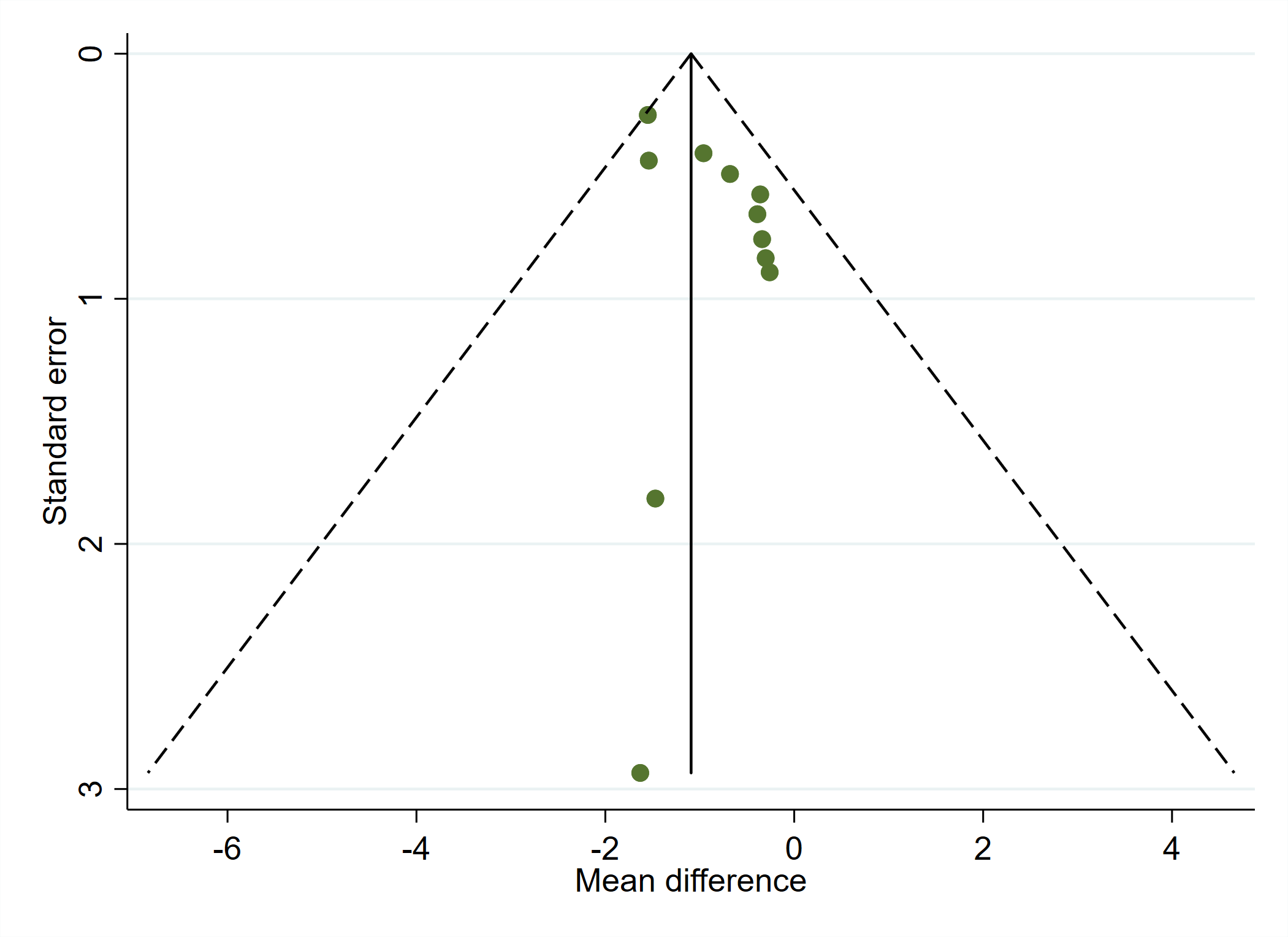

Supplement: Supplementary file 1 [file jcm-13-01323-s001.zip › Supplementary Figure S4.tif]

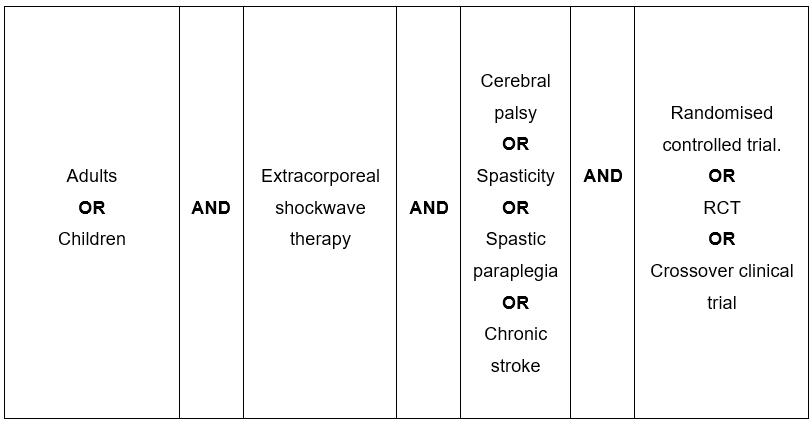

Supplement: Supplementary file 1 [file jcm-13-01323-s001.zip › Supplementary Table S1.tif]

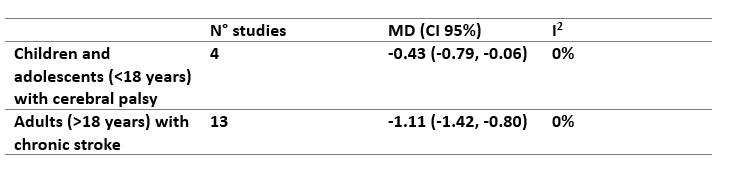

Supplement: Supplementary file 1 [file jcm-13-01323-s001.zip › Supplementary Table S2.tif]

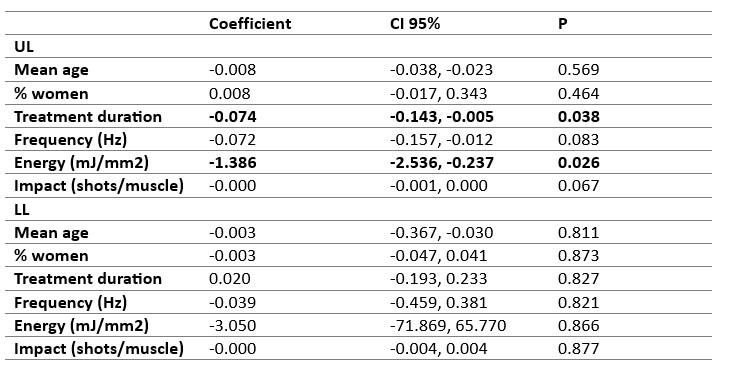

Supplement: Supplementary file 1 [file jcm-13-01323-s001.zip › Supplementary Table S3.tif]

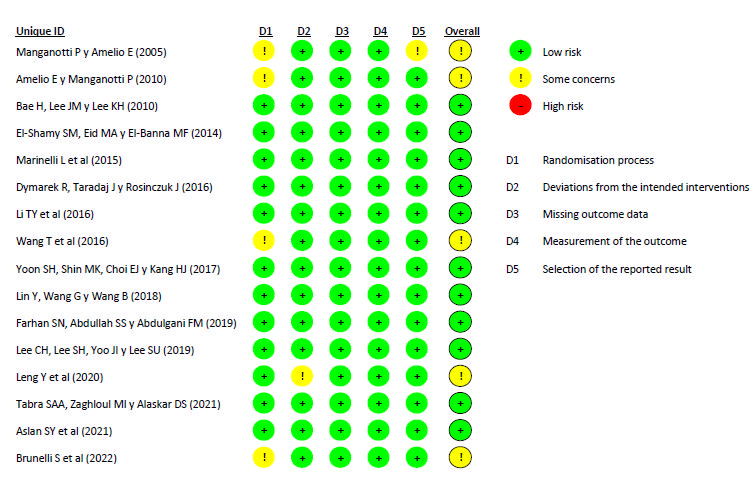

Supplement: Supplementary file 1 [file jcm-13-01323-s001.zip › Supplementary Figure S1.tif]
